# Possible depression in new tuberculosis patients in the Free State province, South Africa

**DOI:** 10.4102/sajid.v39i1.653

**Published:** 2024-08-30

**Authors:** Gladys Kigozi-Male, Christo Heunis, Michelle Engelbrecht, Raymond Tweheyo

**Affiliations:** 1 Centre for Health Systems Research and Development, Faculty of the Humanities, University of the Free State, Bloemfontein, South Africa; 2 Department of Health Policy Planning and Management, School of Public Health, Makerere University Kampala, Uganda

**Keywords:** tuberculosis, depression, comorbidity, prevalence, Free State province, primary health care

## Abstract

**Background:**

Despite compelling evidence of comorbidity between tuberculosis (TB) and depression, little is known about the prevalence and determinants of depression among TB patients in the Free State province in South Africa.

**Objectives:**

This study assessed the prevalence and factors associated with possible depression among new drug susceptible TB patients attending primary health care facilities.

**Method:**

The study followed a cross-sectional design. Trained fieldworkers conducted face-to-face interviews with conveniently selected patients. Depression was assessed using the Patient Health Questionnaire-9. Data were subjected to descriptive and binomial logistic regression analyses.

**Results:**

Out of 208 patients, 46.2% screened positive for possible depression – 22.6%, 18.8%, and 4.8% presenting with mild, moderate, and severe symptoms, respectively. Possible depression odds were three times higher among females than males (adjusted odds ratio [AOR]: 3.0; 95% confidence interval [CI]: 1.25–7.32) and 2.7 times higher among extrapulmonary TB (EPTB) than pulmonary TB patients (95% CI: 1.03–7.21). Longer TB treatment duration was protective (AOR: 0.8; 95% CI: 0.70–0.95) against depression. Among human immunodeficiency virus-positive patients, those on antiretroviral therapy (ART) had 2.5 times higher odds of depression (95% CI: 1.13–5.46) than those who were not.

**Conclusion:**

The results highlight a significant burden of possible depression among new TB patients, particularly among females, EPTB patients, and ART recipients. Longer TB treatment duration may offer some protection against depression symptoms, suggesting a need for enhanced adherence support.

**Contribution:**

The results suggest that strengthening TB and mental health service integration is critical to improving treatment outcomes, overall well-being of TB patients, and the performance of the Free State TB programme.

## Introduction

In 2022, approximately 10.6 million people worldwide contracted tuberculosis (TB). Despite a 13.5% decline in cases, the TB incidence rate in South Africa was 468 cases per 100 000 population, well short of the targeted 50% reduction by 2025.^1^ South Africa reported 280 000 new TB cases, remaining among the 30 high TB burden countries contributing 87% of global cases.^1^ Subpar TB treatment success and high TB mortality rates have remained areas of continued public health concern for more than two decades. With treatment coverage of 77%, treatment success of 79%, and 54 000 deaths registered in 2022,^1^ South Africa is sorely challenged to attain the national target of ensuring that at least 90% of all people diagnosed with TB are successfully treated.^1,2^

TB treatment often complicates with comorbid mental health issues, including depression, anxiety, and psychosis.^3,4^ A 2020 review in low- and middle-income countries (LMICs) found up to 84% depression prevalence among TB patients.^4^ Another study^5^ conducted in South Africa’s Eastern Cape province reported that 8 in 10 TB patients screened positive for at least one psychiatric disorder, including depression and anxiety. Much higher rates of depression are seen in TB populations compared to general populations.^6,7^

Although depression occurs more frequently among TB patients than the general population, it often remains undiagnosed or misdiagnosed^8,9,10,11^ because of various factors. For instance, limited access to mental healthcare services, especially in LMICs such as South Africa;^12^ healthcare providers not prioritising mental health issues;^10^ insufficient mental health service resources;^10,11,12^ stigma surrounding mental health may deter patients from discussing their issues;^13,14^ and overlapping symptoms between TB and depression symptoms can complicate diagnosis.^15^

The interplay between TB and depression is quite complex. Both conditions share parallel risk factors such as poverty, malnutrition, compromised immune functions, and social stigma.^15^ To determine which condition came first becomes challenging because of these bi-directional risk factors.^8,15^ The co-occurrence of TB and depression, compounded by social and economic disparities, hinders healthcare access and worsens outcomes.^15^ Besides, depression increases the risk of latent TB progressing to active disease.^16,17^ Depression may also occur during TB treatment,^5,18^ patients may encounter a broad spectrum of mental health distress, which can range from transient negative emotions, which tend to decrease during the course of TB treatment, to chronic depression characterised by suicide ideation, which may persist beyond the TB period.^10^ In addition, comorbid depression may aggravate TB treatment non-adherence, increasing the risk of unsuccessful outcomes.^19^ Furthermore, the side effects of the anti-TB medication may also contribute to depression.^16,17,19^ To improve the TB programme performance, it is imperative to understand the magnitude of depression, identify its risk factors, and devise ways to manage depression in TB patients optimally.

Research conducted in 2018 on the state of mental healthcare service collaboration in the Free State province of South Africa identified limited screening for mental health conditions at primary health care (PHC) facilities, with severe mental health conditions, referred upwards to district and regional hospitals.^20^ However, little is known about the prevalence and determinants of depression among new TB patients presenting at PHC facilities in the Free State. Therefore, our study sought to examine the prevalence and factors associated with possible depression among patients attending selected PHC facilities in this province.

## Research design and methods

### Design and setting

The study followed a cross-sectional design. Data were gathered among TB patients across 11 PHC facilities in the Free State province from November 2019 to December of 2019. The Free State, with 10 994 new TB cases and a mortality rate of 10.6% in 2019 – the highest among the nine South African provinces^21^ – was purposefully selected for the study. Despite prevailing calls for integration, health services at the PHC level in this province are largely verticalised.^22,23^ The National Mental Health Policy Framework has only recently endorsed including mental health data elements in the District Health Information System.^24^ Prior to this, mental health statistics were not routinely captured and considered by the TB programme.

### Participants

Given the constraint of limited resources, data collection was undertaken over one month, during which 208 patients were conveniently interviewed. Patients were eligible for the study if they were 18 years or older, on susceptible TB treatment, and could express themselves in English or Sesotho. Patients who were too ill to be interviewed and those who were already diagnosed with mental health conditions at the time of the research visit were excluded from the study. Patients undergoing TB re-treatment and those diagnosed with drug-resistant TB were excluded from the study as they tend to present with more severe and chronic TB disease involving complex treatment regimens and frequent hospitalisation.

To ensure that this study had sufficient statistical power (at least 80%), the G–Power calculator for sample size estimation was utilised.^25^ In the analysis utilising sex as a predictor variable and given the preponderance of depression among females,^8^ it was assumed that 50% of women and 30% of men would exhibit symptoms of depression, with an odds ratio (OR) of 2.33 between the two groups. Considering a desired margin of error of 0.05, the calculation indicated that a minimum sample size of 190 patients would be necessary for a robust binomial logistic regression analysis. This sample would ensure that the study was adequately powered to provide reliable estimates.

### Instrument and data collection

A structured fieldworker-administered questionnaire was used to collect the data. Socio-demographic questions included age, sex, and social support. With three questions, the Oslo Social Support Scale (OSSS-3)^26^ was used to measure patients’ perceptions of social support: Firstly, ‘How many people are so close to you that you can count on them if you have great personal problems?’ Response options included: 1 = ‘none’, 2 = ‘1 to 2 people’, 3 = ‘3 to 5 people’, and 4 = ‘more than 5 people’. Secondly, ‘How much interest and concern do people show in what you do?’ Response options included: 1 = ‘none’, 2 = ‘little’, 3 = ‘uncertain’, 4 = ‘some’, and 5 = ‘a lot’. Thirdly, ‘How easy is it to get practical help from neighbours?’ Response options included: 1 = ‘very difficult’, 2 = ‘difficult’, 3 = ‘possible’, 4 = ‘easy’, and 5 = ‘very easy’. The OSSS-3 has demonstrated a somewhat acceptable internal consistency of 0.6, primarily attributed to its brevity.^26^ Despite this, the scale is widely used in diverse research settings because of its conciseness and ease of administration.^26^

Possible depression was measured using the nine-item Patient Health Questionnaire (PHQ-9).^27,28^ The scale assessed the frequency of depressed mood during the two weeks before the research visit. Responses were recorded on a 4-point Likert scale: 0 = ‘not at all’, 1 = ‘several days’, 2 = ‘more than half the days’, and 3 = ‘nearly every day’. Adjustments were made to the response set to enhance respondent comprehension such that ‘several days’ was defined as ‘1 to 7 days’, ‘half the days’ was defined as ‘8 to 11 days’, and ‘nearly every day’ was defined as ‘12 to 14 days’. The PHQ-9 has been found to be valid and reliable for use among TB patients in South Africa.^29^ Clinical information extracted from patient records at the PHC facilities included TB disease classification, treatment duration, alcohol use, tobacco use, human immunodeficiency virus (HIV) status, and antiretroviral treatment (ART) status.

The questionnaire and consent form were available in English and Sesotho. A bilingual translator performed a forward translation of the research instruments from English into Sesotho. Then, another bilingual translator translated the Sesotho versions back into English. Thereafter, both translators compared the back-translated versions to the original versions of the research instruments, engaging in discussions to resolve any emerging discrepancies and reach a consensus.

A team of experienced bilingual fieldworkers, proficient in Sesotho and English, conducted face-to-face interviews in the patients’ preferred language. Participants were recruited with the assistance of healthcare providers. After their consultation session, the attending nurse referred eligible patients to trained fieldworkers waiting in private rooms within the PHC facility. The fieldworkers were trained to look for signs of distress and refer patients to a social worker or psychologist attached to the PHC facility for further assistance. The fieldworkers scored the patients on the PHQ-9 immediately after the interviews. Patients whose PHQ-9 scores suggested possible depression (≥ 5)^27,28^ were referred to the social workers or psychologists for further evaluation and diagnosis. The questionnaire took approximately 10 minutes to complete.

### Data analysis

Data were analysed using the IBM Statistical Package for the Social Sciences (SPSS) statistics (version 27).^30^ Discrete variables were presented as frequency counts and percentages, and continuous variables were presented as means (standard deviations [s.d.]) and medians (inter-quartile range [IQR]). Chi-square tests of independence were used to assess whether there were significant differences between categorical variables. Sum scores were computed for the OSSS-3, ranging from 3 to 14. Higher scores represented more robust levels of social support.^26^ Similarly, sum scores were computed for the PHQ-9, with scores above 4 suggesting possible depression.^27,28^ Regarding the grading of symptoms based on the PHQ-9, total scores falling between 0 and 4 suggested minimal symptoms, scores ranging between 5 and 9 suggested mild symptoms, scores between 10 and 14 suggested moderate symptoms, and scores falling between 15 and 27 suggested severe symptoms.^27,28^

Binomial logistic regression was performed to ascertain the factors associated with possible depression. The outcome variable was possible depression, defined as a score of ≥ 5 on the PHQ-9. The following key assumptions for the binomial logistic regression were inspected. Firstly, the independence of observations was examined using plots of standardised residuals and predicted values, and a random scatter of points around zero was demonstrated, suggesting independence. Secondly, the linearity of the logit of continuous variables was inspected using the Box Tidwell procedure. Based on this assessment, all continuous variables were linearly related to the logit transformation of the dependent variable. Thirdly, the occurrence of extreme outliers examined through inspection of studentised residuals identified two potential outliers with values greater than 2, while the inspection of the leverage values identified five high-leverage values. Since these unusual points were not because of data entry errors, the cases were maintained in the analysis. Fourthly, multicollinearity was assessed using a simple correlation analysis among continuous variables. The analysis established that the correlation coefficients were all less than 0.7, suggesting an absence of multicollinearity.

A test of the full model against a constant-only model was statistically significant (χ^2^ = 26.410, *p* < 0.001, degrees of freedom [*df*] = 9), indicating that the predictors, as a set, reliably distinguished between possible depression and the absence of symptoms. The model explained 23.5% (Nagelkerke’s *R*^2^) of the variance of possible depression. The overall prediction success was 69.9%, with a specificity of 65.6% and a sensitivity of 73.6%. The positive predictive value was 68.9%, and the negative predictive value was 70.7%. Statistical significance was considered at a 95% confidence interval (CI) and *p* < 0.05.

### Ethical considerations

Ethical approval for this study was received from the Health Sciences Research Ethics Committee, University of the Free State (UFS-HSD2019/1574/2611). The Free State Department of Health authorised access to PHC facilities to interview TB patients. Participation in the study was voluntary; prospective respondents were provided with information regarding the purpose of the research and potential risks to enable them to make an informed choice on whether to participate. Respondents signed informed consent forms consenting to the interviews and for their clinical information to be collected from the patient files. The paper-based data were captured in SPSS and secured in encrypted files on a password-protected computer. All data-gathering instruments, including informed consent forms and questionnaires, were kept in locked cabinets. If patients experienced emotional discomfort or distress during the interviews, or their PHQ-9 scores were higher than 4, suggesting possible depression, they were referred to a social worker or psychologist attached to the PHC facility for further assistance. Furthermore, all participants’ information was handled confidentially. The research team was required to sign statements agreeing to protect the security and confidentiality of participants’ information. Patients’ names and other identifying information were collected to verify their clinical information. However, the data were aggregated for dissemination. Efforts were made to ensure infection control during the data-gathering exercise. The patient interviews occurred in privately situated and well-ventilated rooms within the PHC facilities.

## Results

### Patients’ socio-demographic and clinical characteristics

Table 1 depicts the socio-demographic and clinical characteristics of the 208 patients interviewed in this study. Most patients were male (*n* = 137, 65.9%). The mean age of the sample was 42.4 (s.d.: 15.2) years and the median age was 38.5 (IQR: 30.3–54.0) years. Most patients were diagnosed with pulmonary TB (PTB) (*n* = 174, 83.7%). The median TB treatment duration was 2.5 (IQR: 1–4) months. More than one-third of the patients were documented in the patient files as, respectively, alcohol (*n* = 81, 38.9%) and tobacco users (*n* = 69, 33.3%). About 6 in every 10 patients were co-infected with HIV (*n* = 118, 56.7%), with the majority (*n* = 84; 71.2%) being recorded in patient files as receiving ART. Just under half (*n* = 96; 46.2%) of the patients in this study screened positive for possible depression with 22.6% (*n* = 47), 18.8% (*n* = 39), and 4.8% (*n* = 10) presenting with mild, moderate, and severe symptoms, respectively. Half (*n* = 59; 50.0%) of the 118 patients co-infected with HIV screened positive for possible depression, with 26.3% (*n* = 31), 17.8% (*n* = 21) and 5.9% (*n* = 7) presenting with mild, moderate and severe symptoms, respectively. While possible depression was higher among TB-HIV coinfected patients than their HIV-negative counterparts, this difference was not statistically significant (χ^2^ = 1.623, *df* = 11; *p* = 0.203).

**TABLE 1:** Patients’ socio-demographic and clinical characteristics (*N* = 208).

Variable	*n*	%	Median	IQR	Mean ± s.d.
**Sex**					
Female	71	34.1	-	-	-
Male	137	65.9	-	-	-
**Age in years**	-	-	38.5	30.3–54.0	42.4 ± 38.5
**Disease classification**					-
EPTB	25	12.6	-	-	-
PTB	174	83.7	-	-	-
**TB treatment duration in months**	-	-	2.51	1–4	-
**Whether patient is using alcohol**					
No	120	57.7	-	-	-
Yes	81	38.9	-	-	-
Not recorded	7	3.4	-	-	-
**Whether patient is using tobacco**					
No	130	62.5	-	-	-
Yes	69	33.2	-	-	-
Not recorded	9	4.3	-	-	-
**Oslo support scale score**	-	-	-	-	10.3 ± 2.32
**HIV status**					
HIV negative	80	38.5	-	-	-
HIV positive	118	56.7	-	-	-
Not recorded	10	4.8	-	-	-
**PHQ-9 symptom severity**					
Minimal symptoms	112	53.8	-	-	-
Mild symptoms	47	22.6	-	-	-
Moderate symptoms	39	18.8	-	-	-
Severe symptoms	10	4.8	-	-	-
**HIV coinfected patients (*N* = 118)**					
Whether patient is on ART					
On ART	84	71.2	-	-	-
Not on ART	23	19.5	-	-	-
Not recorded	11	9.3	-	-	-
PHQ-9 symptom severity					
Minimal symptoms	59	50.0	-	-	-
Mild symptoms	31	26.3	-	-	-
Moderate symptoms	21	17.8	-	-	-
Severe symptoms	10	5.9	-	-	-

ART, antiretroviral therapy; EPTB, extrapulmonary tuberculosis; HIV, human immunodeficiency virus; IQR, inter-quartile range; PHQ-9, patient health questionnaire-9; PTB, pulmonary tuberculosis; s.d., standard deviation.

### Factors associated with possible depression

Binomial logistic regression was performed to determine the effect of sex, age, TB disease classification, treatment duration, social support, tobacco and alcohol use, and HIV status on possible depression. The results in Table 2 depict that sex (OR: 3.2; CI: 1.76–5.83) and treatment duration (OR: 0.8; CI: 0.73–0.97) were independently significantly associated with possible depression. However, because of their theoretical association with the dependent variable, all the independent variables were considered in the adjusted model.^31^ After controlling for all variables in the model, possible depression was significantly associated with sex, TB disease classification, and treatment duration. Female patients were 3 times more likely to present with possible depression compared to their male counterparts (AOR: 3.0; CI: 1.25–7.32). Patients diagnosed with extra pulmonary TB (EPTB) were 2.7 times more likely to present with possible depression compared to those diagnosed with PTB (CI: 1.03–7.21). Alternatively, longer TB treatment was protective against possible depression (AOR: 0.8; CI: 0.73–0.95). Furthermore, Table 3 shows that after adjusting for confounding variables, the likelihood of possible depression was higher among HIV coinfected females (AOR: 2.8; CI: 1.22–6.56), EPTB patients (AOR: 3.5; CI: 1.11–11.50), and patients who were receiving ART (AOR: 2.5; CI: 1.13–5.46) relative to their respective counterparts, and less likely among those on TB treatment for longer (AOR: 0.8; CI: 0.67–0.97).

**TABLE 2:** Factors associated with possible depression among all TB patients (*N* = 208).

Variable	Possible depression (*n* = 96)								*p*
	*n*	%	Mean	s.d.	Unadjusted OR	CI	Adjusted OR	CI	
**Sex**	-	-	-	-	-	-	-	-	0.002
Male (ref)	50	52.10	-	-	1.0	-	1.0	-	-
Female	46	47.90	-	-	3.2	1.76–5.83	3.0	1.25–7.32	-
**Disease classification (*n* = 91)**	-	-	-	-	-	-	-	-	0.044
PTB (ref)	75	82.40	-	-	1.0	-	1.0	-	-
EPTB	16	17.60	-	-	2.4	0.98–5.60	2.7	1.03–7.21	-
**Treatment duration in months**			2.1	1.90	0.8	0.73–0.97	0.8	0.70–0.95	0.008
**Whether patient is using alcohol**	-	-	-	-	-	-	-	-	0.763
No/not recorded (ref)	73	76.00	-	-	1.0	-	1.0	-	-
Yes	23	24.00	-	-	0.8	0.43–1.33	0.7	0.32–1.36	-
**Whether patient is using tobacco**	-	-	-	-	-	-	-	-	0.244
No/not recorded (ref)	66	68.70	-	-	1.0	-	1.0	0.73–3.44	-
Yes	30	31.30	-	-	0.9	0.48–1.52	1.6	-	-
**Oslo support score**			10.0	2.46	0.9	0.80–1.02	0.9	0.80–1.05	0.232
**HIV status**	-	-	-	-	-	-	-	-	0.376
Negative/not recorded (ref)	13	22.00	-	-	1.0	-	1.0	-	-
HIV-positive	46	78.00	-	-	1.4	0.82–2.49	1.3	0.71–2.51	-

CI, confidence interval; EPTB, extrapulmonary tuberculosis; HIV, human immunodeficiency virus; OR, odds ratio; PTB, pulmonary tuberculosis; ref, reference group; s.d., standard deviation; TB, tuberculosis.

**TABLE 3:** Factors associated with possible depression among TB-HIV coinfected patients (*N* = 118).

Variable	Possible depression (*n* = 59)								*p*
	*n*	%	Mean	s.d.	Unadjusted OR	CI	Adjusted OR	CI	
**Sex**	-	-	-	-	-	-	-	-	0.015
Male (ref)	28	47.50	-	-	1.0	-	1.0	-	-
Female	31	52.50	-	-	2.3	1.10–4.92	2.8	1.22–6.56	-
**Disease classification (*n* = 57)**	-	-	-	-	-	-	-	-	0.033
PTB (ref)	43	75.40	-	-	1.0	-	1.0	-	-
EPTB	14	24.60	-	-	3.4	1.13–10.15	3.5	1.11–11.51	-
**Treatment duration in months (*n* = 115)**			2.7	2.10	0.9	0.72–1.03	0.8	0.67–0.97	0.027
**Whether patient is using alcohol**	-	-	-	-	-	-	-	-	0.763
No/not recorded (ref)	38	64.40	-	-	1.0	-	1.0	1	-
Yes	21	35.60	-	-	0.8	0.36–1.58	0.7	0.35–2.15	-
**Whether patient is using tobacco**	-	-	-	-	-	-	-	-	0.677
No/not recorded (ref)	37	62.70	-	-	1.0	-	1.0	1	-
Yes	22	37.30	-	-	0.6	0.29–1.36	1.2	0.45–3.37	-
**Oslo support score**			10.4	2.40	0.9	0.80–1.05	0.9	0.80–1.10	0.191
**Whether patient is receiving ART**	-	-	-	-	-	-	-	-	0.023
No/not recorded (ref)	13	22.00	-	-	1.0	-	1.0	-	-
On ART	46	78.00	-	-	2.0	0.87–4.41	2.5	1.13–5.46	-

ART, antiretroviral therapy; CI, confidence interval; EPTB, extrapulmonary tuberculosis; HIV, human immunodeficiency syndrome; OR, odds ratio; PTB, pulmonary tuberculosis; s.d., standard deviation.

## Discussion

This study analysed possible depression prevalence and associated factors among new patients on susceptible TB treatment in the Free State province. Nearly half (46.2%) of the TB patients screened positive for possible depression. TB-HIV coinfected patients showed higher levels of possible depression relative to their non-HIV coinfected counterparts, although not statistically significant. Possible depression prevalence was slightly lower than in an Ethiopian study (54%)^8^ possibly because of the demographic and clinical differences. Unlike the Ethiopian study, this study included patients at any stage of TB treatment, not just within 1 month.

Different levels of TB and depression comorbidity have been documented across the studies conducted on the African continent over the past decade, ranging from a relatively low prevalence of 9.3% in Zambia^32^ to a notably high prevalence of 61.0% in Cameroon.^33^ A systematic review of sub-Saharan African studies spanning from 1992 to 2019 further reported a pooled depression prevalence of 39.4% among TB patients.^34^ While no comparisons were made with general (non-TB) patients, the prevalence of possible depression in this study was slightly higher than the values reported in other populations such as patients with epilepsy (32.7%)^35^ and pregnant women with no specific condition (22.8%).^36^ This study’s finding adds to the expanding body of literature substantiating the intricate interplay between TB and mental health disorders and underscores an urgent need for a paradigm shift in service delivery at the PHC level. More specifically, it is imperative to enhance the integration of mental health services into the TB programme to ensure the timely identification and management of mental health conditions such as depression among TB patients, mitigate the stigma associated with mental illness, and strengthen the overall effectiveness of TB programmes. In line with this imperative, the recently unveiled National Mental Health Policy Framework and Strategic Plan highlights, among other efforts, the need for ongoing mental health training for PHC nurses.^24^

Consistent with global research,^8,37,38,39,40,41^ this study revealed that female TB patients had about 3 times higher odds of possible depression than males, regardless of HIV status. Future research could investigate whether this gender disparity persists in TB patients. This would inform whether TB programmes should consider gender-specific interventions, particularly mental health screening services, care, and management targeting female patients and their support systems.

Similar to research in Ethiopia,^40^ possible depression was more pronounced among EPTB than PTB patients, which could potentially be attributed to the difficulty in diagnosis, intense treatment regimens, and the often poor prognosis associated with EPTB.^42,43^ EPTB has distinct manifestations with symptoms that may not be as widely recognised as those of PTB.^42^ Thus, the lack of awareness may contribute to patients experiencing stigma and social isolation, potentially affecting their mental health.

Analysis of the HIV coinfected sub-group showed that patients on ART were nearly 3 times more likely to experience possible depression relative to those not receiving ART. Both TB and HIV involve treatment using complex regimens and side effects^44,45^ associated with these treatments may lead to psychological distress. Thus, TB programmes could explore practical methods for the timely identification and treatment of patients with common mental health problems such as depression. In resource-limited settings such as South Africa, with an accompanying shortage of professional mental health practitioners at the PHC level, using non-mental health specialists such as community health workers^46,47,48^ could prove highly beneficial for the targeted screening of patients within TB programmes, especially if supplemented with adequate ongoing training, support, supervision and monitoring.^47^

Similar to findings in Ethiopia,^41^ longer TB treatment duration was associated with reduced depressive symptoms. Given that a substantial proportion of the patients in our study had mild to moderate depressive symptoms, their symptoms might have improved, and they were potentially feeling better the longer they were on TB treatment. Alternatively, the patients could possibly have gained a better understanding of TB over time, subsequently developing more effective ways to cope with the psychological challenges posed by the disease.

While socio-economic status can significantly affect mental health outcomes,^49,50^ this study did not establish an association between socio-economic variables such as age, social support, alcohol and tobacco use, and the likelihood of possible depression. Further research is recommended to explore the relationship between socio-economic status and mental health outcomes among TB patients.

This study has some limitations. Firstly, the results were based on a convenient sample of patients and cannot be generalised to all TB patients in the Free State. Secondly, only those patients who were present on the day of the research visit and were well enough to participate were interviewed. There is a possibility that more severely depressed patients were missed, which might have underrepresented the prevalence of possible depression. Thirdly, as the data collection was based on fieldworker-administered questionnaires, some patients might have been inclined to provide socially desirable responses while speaking to the fieldworkers. Fourthly, while clinical information was derived from patient records at the PHC facilities, likely from clinician’s notes, it was not corroborated by laboratory testing or pharmacy dispensing records, or validated screening tools. Nonetheless, the study’s findings offer valuable insights about TB-depression comorbidity that can guide further research and the development of targeted interventions and policies in the future in the Free State and similar settings.

## Conclusion

This study highlights a significant burden of possible depression among new TB patients, respectively, estimated at 46.2% among all new TB patients and 50.0% among TB-HIV coinfected patients attending certain PHC facilities in the Free State. The results suggest the need to strengthen integrated TB and mental health service delivery in the province. Patients at high risk of depression, including females, EPTB patients, and TB-HIV-coinfected patients on ART, should be prioritised for mental health screening and provided with adequate psychosocial support. As TB treatment duration seems to give a degree of protection against depressive symptoms, more treatment support is warranted within the care cascade. These are essential steps towards improving treatment outcomes, TB patients’ overall well-being, and the performance of the TB programme in the Free State and similar settings.
